# Analysis of Phenolic and Cyclic Compounds in Plants Using Derivatization Techniques in Combination with GC-MS-Based Metabolite Profiling

**DOI:** 10.3390/molecules20023431

**Published:** 2015-02-17

**Authors:** Jens Rohloff

**Affiliations:** Department of Biology, Norwegian University of Science and Technology, Trondheim 7491, Norway; E-Mail: jens.rohloff@bio.ntnu.no; Tel.: +47-7359-6093; Fax: +47-7359-6100

**Keywords:** derivatization, food chemistry, gas chromatography, mass spectrometry, phenols, phenolic acids

## Abstract

Metabolite profiling has been established as a modern technology platform for the description of complex chemical matrices and compound identification in biological samples. Gas chromatography coupled with mass spectrometry (GC-MS) in particular is a fast and accurate method widely applied in diagnostics, functional genomics and for screening purposes. Following solvent extraction and derivatization, hundreds of metabolites from different chemical groups can be characterized in one analytical run. Besides sugars, acids, and polyols, diverse phenolic and other cyclic metabolites can be efficiently detected by metabolite profiling. The review describes own results from plant research to exemplify the applicability of GC-MS profiling and concurrent detection and identification of phenolics and other cyclic structures.

## 1. Introduction

Chromatographic techniques for the detection and identification of metabolites in plant material have undergone major changes in recent years due to improvements of analysis time, detection limit and separation characteristics. Depending on the biological question, one might distinguish between targeted and non-targeted strategies. Gas chromatography (GC) in particular is characterized by sensitivity and reliability of separations and detection of complex sample mixtures. Coupling with mass spectrometry (MS) provides highly robust analysis platforms compared to liquid chromatography (LC-MS) and allows for the identification of compounds based on the use of commercially or publicly available MS libraries and resources (Table 1) in combination with retention time index (RI) data.

**Table 1:** Selection of commercially and publicly available MS libraries and resources for structure elucidation and compound identification of GC-MS data. Included is also a list of freely software tools for identification, deconvolution and alignment purposes.

Product	Supplier or Institution
Commercial MS Libraries	
**NIST**—NIST/EPA/NIH Mass Spectral Library	National Institute of Standards and Technology/Gaithersburg, MD, USA
**Wiley**—Wiley Registry of Mass Spectral Data	John Wiley & Sons, Inc./ Hoboken, NJ, USA
**FiehnLib**—Fiehn GC-MS Metabolomics RTL Library	Agilent Technologies, Inc./ Santa Clara, CA, USA
Public MS Libraries & Resources	
**GMD**—Golm Metabolome Database	Max Planck Institute of Molecular Plant Physiology/Golm, Potsdam, Germany
**MassBank**—High Quality Mass Spectral Database	National Bioscience Database Center/Tokyo, Japan
**MetabolomeExpress**—Public MSRI Libraries	Plant Energy Biology, ARC Centre of Excellence/Acton, Canberra, Australia
**ReSpect**—Riken MSn spectral database (LC/MS)	Metabolomics Research Division, RIKEN Plant Science Center,/Tsuruoka, Japan
**Metlin**—Metabolite and Tandem MS Database	Scripps Center for Metabolomics/La Jolla, CA, USA
**HMDB**—Human Metabolome Database	Genome Alberta & Genome Canada, University of Alberta/Edmonton, Canada
***m/z* CLOUD**—Advanced Mass Spectral Database	HighChem Ltd. / Bratislava, Slovakia
**NIST**—NIST Chemistry WebBook	National Institute of Standards and Technology/Gaithersburg, MD, USA
Free GC/MS Analysis Software & Tools	
**AMDIS**—Automated Mass Spectral Deconvolution and Identification System	National Institute of Standards and Technology/Gaithersburg, MD, USA
**Tagfinder**—GC-MS analysis software (free upon request)	Max Planck Institute of Molecular Plant Physiology/Golm, Potsdam, Germany
**MetaboliteDetector**—Data deconvolution & analysis	TU Braunschweig, Germany
**OpenChrom**—Software for chromatography and MS	Dr. Philip Wenig/ Hamburg, Germany
Free GC/MS Alignment Tools	
**Metalign**—Processing of LC-MS and GC-MS data	Wageningen UR (University & Research centre)/Wageningen, The Netherlands
**MZmine**—Processing of LC-MS and GC-MS data	Turku Centre for Biotechnology/ Turku, Finland
**MetaboAnalyst**—Comprehensive tool suite for metabolomic data analysis	The Metabolomics Innovation Centre (TMIC)/University of Alberta, Canada
**SpectConnect**—GC-MS data alignment and analysis	Massachusetts Institute of Technology (MIT)/Boston, MA, USA

Recent technological advances in high-resolution and high-throughput methods generally termed as *metabolomics*, allow for large scale GC-MS profiling regarding the high number of measured metabolites and experiments carried out [1]. Gas chromatography time-of-flight mass spectrometry (GC-TOF-MS) provides fast scanning, high sensitivity and mass accuracy compared to common quadrupole (GC-QMS) or ion trap instrumentation (GC-ITMS), and is considered the standard GC platform in many metabolomics labs. Sample processing for GC-MS-based metabolite profiling include solvent extraction, concentration to dryness and consecutive derivatization, often carried out in a two-step procedure. In the first step, methoximation (also called methoxyamination) is achieved by a reaction of sample components with e.g., *O*-methoxylamine hydrochloride diluted in pyridine to stabilize thermolabile enolic aldehydes and ketones and to convert them into oximes or alkyl oximes. In the second step, extracted metabolites are derivatized with silylating reagents [1,2]. The latter step is crucial for the adequate derivatization of non-volatile compounds, in order to capture a huge variety of metabolites with polar characteristics and high boiling points on a GC-MS system. Detectable compounds comprise sugars (mono-, di- and trisaccharides), sugar alcohols/acids, amino and fatty acids, phosphorylated intermediates and many plant secondary metabolites such as phenolics, terpenoids, steroids and alkaloids.

These efforts have finally led to the development of searchable databases and libraries such as the Golm Metabolome Database (GMD) and the MassBank MS Database, which provide mass spectral information about TMS derivatives (Table 1). In addition to instrument-based GC-MS software (e.g., Agilent ChemStation and Thermo Scientific’s Mass Frontier), a wide range of deconvolution and analysis programmes are freely available, either bundled with MS libraries such as AMDIS (NIST MS library) and Tagfinder (GMD), or stand-alone software such as MetaboliteDetector and OpenChrom. Consecutive alignment of vast numbers of sample files from large-scale experiments is an indispensable procedure generating huge output matrices of thousands or even millions of data points. The processing of single fragment ion information is a common feature of advanced GC-MS alignment tools. It further allows for the identification of conserved compounds and metabolite patterns present in all samples, or unique metabolite markers which can be traced based on characteristic ion fragments. However, the use of reference substances, and if not available, corresponding scientific literature and online tools are often inevitable, in order to receive information about molecular ions (M^+^) and common fragments for the detection of molecules of interest. The plant kingdom produces a vast number of different chemical structures, which is predicted to exceed 200,000 metabolites [3]. Moreover, MS information of silylated natural products is insufficiently represented in available compound libraries for GC-MS platforms based on electron ionization (EI). More suitable chromatographic profiling platforms such as LC-MS are available for analysis of metabolites with higher polarity and molecular weights up to 2000 Da [4]. In addition, comprehensive mass spectral libraries (MS, MSn data) containing MS information about thousands of secondary structures, are readily accessible for compound identification, e.g., ReSpect for Phytochemicals, Metlin Metabolite and Tandem MS Database, and MassBank. However, GC-MS-based metabolite profiling of derivatized polar extracts is capable of capturing a huge variety of smaller secondary metabolites up to 800 Da as presented in the following sections.

## 2. GC-MS Profiling of Complex Chemical Matrices

Beside parameters related to proper MS compound identification and use of retention index (RI) data, several aspects related to metabolite extraction and derivatization need to be considered in GC-MS-based metabolite profiling. Depending on the biological question, metabolite targets, and sample matrix, solvents of differing polarity and varying phases have been investigated for compound extraction. For global metabolomics approaches, the use of extraction mixtures covering a wide range from polar to apolar metabolites such as H_2_O:MeOH:CHCl_3_ (1:2.5:1) or H_2_O:MeOH are favoured [2,5]. In other cases, one might need to separate lipids from the polar phase as described by Lisec *et al*. [1], either for separate chromatographic study of the different fractions [6], or for a more targeted metabolite analysis.

### 2.1. Extraction Methods and Metabolite Coverage

While sample preparation and processing need to be individually adapted and customized, sample extraction often follows standardized procedures using established protocols for comprehensive metabolite profiling and non-targeted metabolomics approaches as already outlined at the beginning. An important parameter in high-throughput metabolite profiling is the high degree of miniaturization and automation in sample handling, requiring the processing of small or ultrasmall sample sizes as low as a few milligrams. Unless secondary metabolites are highly abundant, their potential recovery under such extraction conditions and GC-MS detection proves insufficient. Moreover, microextraction techniques such as sorbent-based solid-phase microextraction (SPME) [7], stirbar-sorptive extraction (SBSE) [8], and solvent-based methods such as e.g., single-drop microextraction (SDME) and liquid-liquid microextraction (LLME) [9] establish sensitive methods for the characterization of complex profiles of volatile compounds found in plant and food samples. Microextraction might also be successfully combined with derivatization techniques and subsequent gas chromatographic separation for the detection of a wide range of volatiles with different polarity [10,11,12]. In general, metabolomic approaches based on SPME extraction and detection of non-derivatized volatiles, emphasize the technique’s tremendous capacity to cover a broad range of different compound groups [13], also including aromatic structures, as shown for e.g., tomato [14] and peach [15].

### 2.2. Derivatization of Metabolites

Derivatization prior to GC-MS is an essential preparatory step, which reduces polarity and increases volatility, and simultaneously, thermal stability of metabolites. Compound derivatization is either based on silylation, alkylation or acylation reactions, and a wide range of reagents with different properties are available. Comprehensive studies have shown the superior properties of silylation agents [16], which substitute protons bound to heteroatoms in functional groups (‑OH, ‑COOH, ‑NH_2_, -NH, -SH, -OP(=O)(OH)_2_, *etc.*) and generate trimethylsilyl (TMS) and *tert-*butyldimethylsilyl (TBS) derivatives [17]. Though *N*-[dimethyl-(2-methyl-2-propyl)silyl]-2,2,2-trifluoro-*N*-methylacetamide (MTBSTFA), *N*,*O*-bis(trimethylsilyl)trifluoroacetamide (BSTFA) and *N*-methyl-*N*-(trimethylsilyl)trifluoroacetamide (MSTFA) are widely applied in biological analyses, the use of MSTFA as derivatization agent has been favoured by leading metabolomics labs worldwide. In specific cases, the use of other derivatization agents might be advised. Newer studies suggest alkylation with e.g., methyl chloroformate (MCF) instead of, or in combination with compound silylation due to improved analytical performance [18]. MCF derivatization shows improved reproducibility and compound stability compared to silylation, and is suitable for the analysis of microbial-derived samples with matrices mainly composed of amino and non-amino organic acids, amines and nucleotides [18]. However, due to the wide range of compound structures being covered by combined methoximation and TMS derivatization [19], comprising amino acids, fatty acids, lipids, amines, alcohols, sugars, amino-sugars, sugar alcohols, sugar acids, organic phosphates, hydroxyl acids, aromatics, purines, and sterols, major metabolomics attempts towards the development of MS libraries have favoured the coverage of oximated and silylated metabolites.

Despite the feasibility and power of combined oximation/silylation in global metabolite profiling approaches, several factors impair sample analysis and data quality. A major point is that silylation reactions have to be carried out under anhydrous reaction conditions [18], which requires an additional drying step of sample extracts (e.g., SpeedVac^TM^ (Thermo Scientific, Waltham, MA, USA) or lyophilization). Excess derivatization reagents are commonly introduced in the GC injection port, potentially leading to additional peaks in the chromatogram. Moreover, also non-volatile non-derivatized metabolites or even macromolecules such as peptides, proteins or polysaccharides might be injected, depending on preceding sample clean-up conditions (precipitation, centrifugation and/or filtration) and thus, impede separation performance and GC data analysis. Nevertheless, the utilization of packed inlet liners and/or suitable guard columns circumvent these problems and can protect analytical capillary columns from sample impurities.

**Figure 1:**
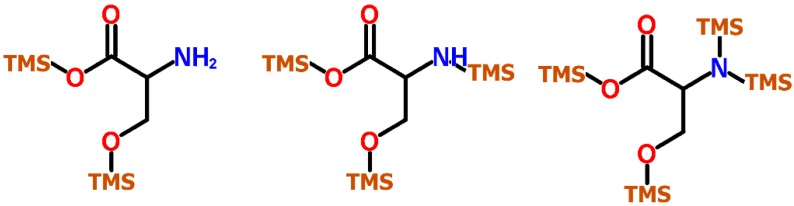
Trimethylsilylation levels of the amino acid serine commonly found in TMS derivatized samples. In serine (2TMS), the OH- and COOH-group are trimethylsilylated, in serine (3TMS) one OH-group of the amino group is exchanged, while in serine (4TMS) all active hydrogens are exchanged.

Another important factor affecting the quality of metabolite data is the occurrence of artefacts of silylated compounds in GC-MS profiling [17]. Unexpected by-products might add to the complexity of peaks in a chromatogram and interfere with the identification process. This includes also conversion reactions of unstable intermediates, e.g., arginine to ornithine when using BSTFA or MSTFA, potentially leading to misinterpretation of metabolic data [4]. But more important, multiple peaks of one and the same metabolite, *i.e.*, with different degree of TMS silylation of the original molecule, might be detected. This is particularly true for those metabolites with several functional groups such as amino acids (-COOH, -NH_2_, -OH) and monosaccharides which carry a high number of hydroxy groups. The amino acid *serine* e.g., shows four active hydrogens which might be exchanged by TMS groups (Figure 1).

In GC-MS one might deal with both the 2TMS stage, where only the carboxy and hydroxy group are silylated, and the 3TMS and 4TMS stage with one or both H^+^ of the amino group exchanged. For this reason, MS data of metabolites showing a varying numbers of TMS groups, and both oximate/ TMS metabolite derivatives have been included in MS libraries (e.g., GMD database, NIST, *etc.*). Such considerations play an important role if metabolites levels shall be quantitatively acquired. Expected that multiple compounds show the same detection response, their individual responses might be summed. Regarding secondary metabolites, it is likely to assume that artifact formation also occurs for these compounds depending on the number of active hydrogens and the given derivatization conditions. However, MS information of such by-products is scarcely, if at all, included in available TMS-MS libraries.

### 2.3. GC-MS Metabolite Profiling—Applications, Performance and Reliability

The term *metabolite profiling* has already existed for many years, but first the development of high-capacity and high-throughput chromatographic systems in recent years has established the basis to generate extensive amounts of profiling data, which is comparable in its extent to the output of proteomics and transcriptomic analyses. Moreover, the applicability and significance of particularly GC-MS metabolite profiling in functional genomics was early recognized using the plant model systems *Arabidopsis thaliana* and potato (*Solanum tuberosum*) [20,21]. Common for those reports is the low coverage of secondary structures which primarily include major phenolic acids, pyridines, tocopherols and sterols. The concept of metabolomics has been applied also for the study of crop plants in more recent years [22]. Using tomato (*Solanum lycopersicum*) as an example, breeding goals towards nutritional quality [23] and yield [24], impact of environment such as fertilization [25], and temporal metabolite patterns in molecular crop physiology have been addressed [26]. Also here, GC-MS metabolite profiling had major focus on central metabolism and less on secondary structures. However, phenolic acids commonly found in plant material have been detected in potato [27,28], maize [29], soybean leaves [30] and tobacco [31]. GC-MS profiling of lipophilic compounds including sterols/triterpenes and tocopherols has been described for tomato cuticles [32] and maize grain [29]. One important and necessary concept for the description of genetically modified (GM) crops is the so-called *substantial equivalence* based on the evaluation whether the chemical composition of a GM crop differs from the non-GM counterpart or not. Metabolite profiling is utilized as an essential tool for screening of GM crops with regard to quality and health requirements in order to investigate potential changes in metabolite profiles in e.g., wheat [33], rice [34], and maize [35].

Global metabolomic approaches based on derivatization techniques following GC-MS for the mapping of biosynthetic pathways and characterization of metabolic perturbations and genotypes appear to be less laborious and more cost-effective compared to metabolite targeting. The use of highly sensitive and fast-scanning GC-TOF-MS instrumentation in particular facilitates proper compound detection and resolution of co-eluting peaks. The latter point is a common feature in comprehensive metabolite matrices, and can successfully be addressed based on the acquired data information, including exact retention time, accurate mass, and characteristic MS fragmentation patterns and ion intensities. In GC-QMS or GC-ITMS, on the other hand, analysis time might be extended in order to improve separation performance and resolution of overlapping peaks. In recent years, more advanced extraction and separation methods have been added to the tool box of automated profiling techniques. Such advances include GC × GC-MS (or so-called 2D GC-MS), where samples are subsequently separated on columns of different polarity, thus increasing separation capacity and performance in the detection of biomolecules [36].

However, even the use of accurate mass, MS spectra and RI values might lead to misidentification of compound structures, not least because mass spectra of different TMS derivatives might be notoriously similar (e.g., sugar alcohols), or totally different metabolites might have the same RI value. Another major problem when analysing complex sample mixtures is the fact that metabolite abundances can vary by many orders of magnitude. The linearity and detector response dynamics of metabolites from different structure groups differ greatly [18], thus impeding quantitation of absolute compound concentrations. For that purpose, levels of distinct metabolites might be determined by comparison with calibration standard curve response ratios of various concentrations of standard substance solutions as described by e.g., Roessner-Tunali *et al.* [37]. Moreover, variations in sample volume, *i.e.*, when using a sample range with a defined maximal tolerance, only allows for relative quantitative detection of metabolites, in contrast to procedures using the same exact amounts of samples and allowing for absolute quantitation [38]—a fact which needs suitable consideration when working with small sample sizes.

### 2.4. Secondary Metabolites in GC-MS-Based Metabolomic Approaches

GC-MS-based metabolite profiling of TMS derivatives does not only generate vast chemical information about primary metabolism, but includes also extensive MS data about secondary metabolites and “unknowns”. In order to advance the identification process of less abundant structures in plant samples, comprehensive RI tables for cinnamic acids and other simple phenolic structures, flavonoids, tocopherols and sterols have been generated to provide useful information about the elution order of silylated secondary metabolites on a GC system [27,39]. Furthermore, MS fragmentation patterns have been reported for a wide range of secondary metabolites including phenols and phenolic acids [40,41,42,43], flavonoids [42,44,45], alkylresorcinols [46], phytoestrogens [47], secoiridoids and ligstrosides [41,48], diterpenes and diterpenic acids [49], phenolic diterpenes and pentacyclic triterpenes [50], sterols, stanols, and esters thereof [51,52], lignans [53], stilbenes [54,55], and alkaloids [56]. The utilization of relevant scientific literature reporting GC-MS information about phytochemicals is crucial for the tentative identification of less abundant chemical structures in profiling experiments. Regarding the restricted number of primary and major secondary metabolites, which are commonly included in spectral libraries of TMS analytes, several specific secondary structures are described based on case studies presented in Section 4.

## 3. Detection of Plant Phenolics and Other Cyclic Structures

The following classification of the quite diverse group of phenolic structures and other plant-derived cyclic compounds follows chemical structure characteristics rather than biosynthetic relationships, which makes it easier to discuss the topic from an analytical point of view. In a wider sense phenolic structures addressed here contain either one (Section 3.1) or several aromatic rings (Section 3.2) as part of the molecule. In order to cope with the tremendous variability of primary but also secondary metabolites detectable by GC-MS, several attempts have been made to facilitate identification through the construction of combined RI and MS databases of derivatized compounds, generally termed as mass spectral tags (MST) [57]. Retention time indices are a prerequisite for tentative metabolite annotation of mass spectra showing high similarity such as pentoses and hexoses. Recent efforts have focused on the need for extended and comprehensive RI information by compiling RI values of TMS analytes of various phytochemicals including phenolic acids and flavonoids [39,45,51,58,59], diterpenes [49], sterols [51] and tocopherols [32].

Moreover, several research groups and consortia have addressed the complexity of compound structures, since commercially available libraries (e.g., NIST or Wiley) contained insufficient MS information about derivatized analytes, which are frequently acquired in metabolomics experiments. The Golm Metabolome Database GMD [60] comprise today online information of about >4600 MS analyte entries including >3500 analytes with valid spectra (TMS derivatives, tributylsilyl (TBS) derivatives, and isotopically-labeled compounds). However more than 1400 spectra have not been annotated underscoring the need for further chemical information in order to approach the metabolome of plants and other organisms. The downloadable and searchable GMD library contains about 3600 analytes and MST information about 1200 single metabolites. In comparison, the Fiehn GC-MS Metabolomics RTL Library [19] is based on 1400 analyte entries relating to 900 metabolites, while the Massbank database [61] contains 963 MSTs of TMS analytes relating to >700 single metabolites. Hopefully, public repositories of GC-MS-based spectral information such as MetabolomeExpress [62] might help to extend accessible MS library information also including secondary metabolites.

Only those phenolic and cyclic structures which are commonly detectable in derivatized (silylated) samples following GC-MS profiling protocols applied by the majority of metabolomics labs worldwide [1,2,63,64], will be discussed in Subsection 3.1, Subsection 3.2, Subsection 3.3, Subsection 3.4, Subsection 3.5 and Subsection 3.6. Information about readily searchable and publicly available MS databases and libraries, providing MS spectra of silylated metabolites, presented and discussed in Section 3 and Section 4, will be included in each figure. The following abbreviations (letters) are used: [G]—Golm Metabolome Database; [H]—Human Metabolome Database; [M]—MassBank; [N]—NIST Chemistry WebBook. For LC-MS-based analysis of phytochemicals in general, the reader is referred to recent studies and initiatives for the development of MS/MS libraries such as the accurate mass-time (AMT) tag approach [65] and the PRIMe platform of RIKEN Plant Science Center [66].

### 3.1. Simple Phenolics, Aromatic Acids and Related Structures

**Figure 2:**
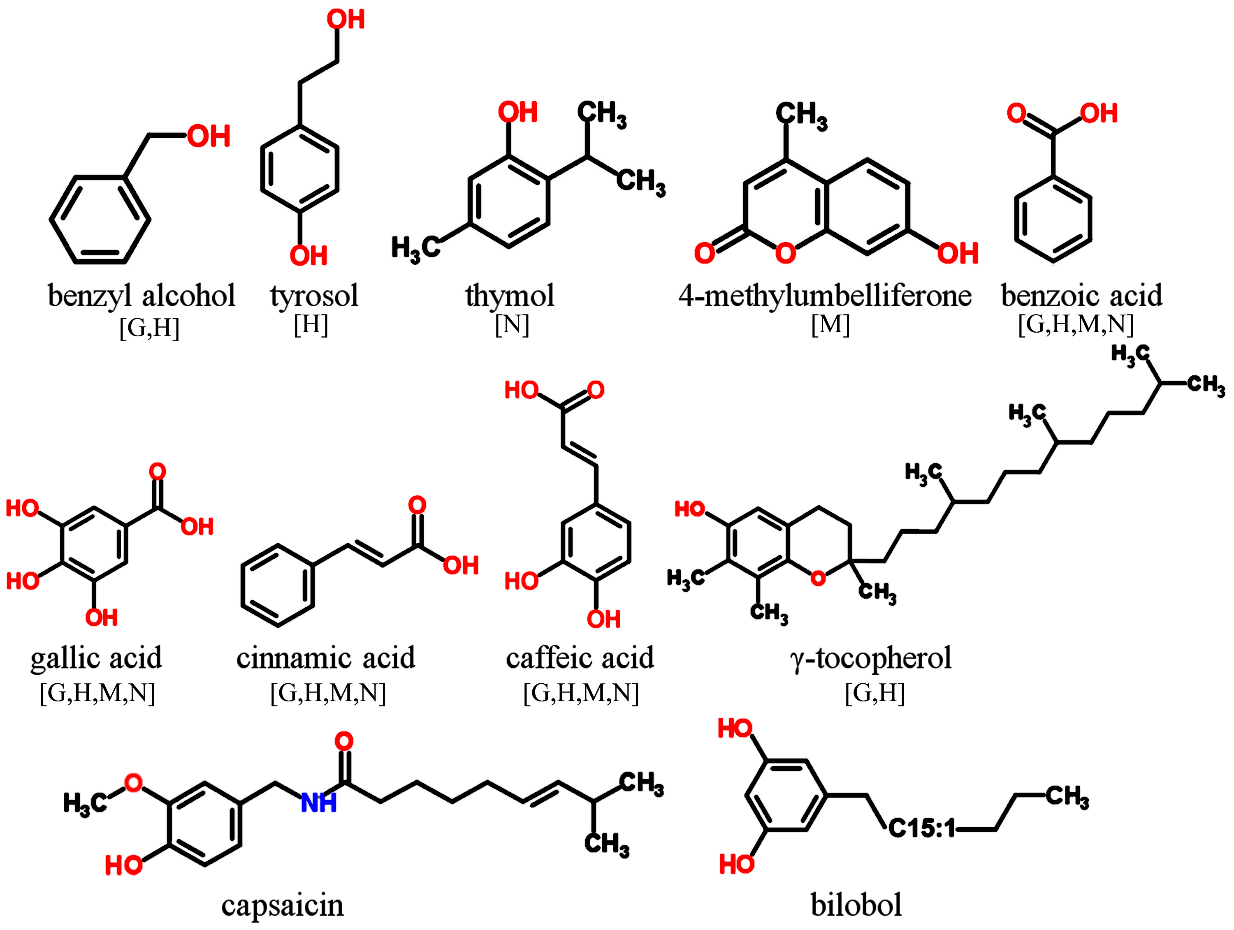
Monophenolics and aromatic acids detectable in GC-MS profiling experiments. MS spectra included in publically available databases.

In most cases phenolic structures are derived from aromatic amino acids such as phenylalanine and tyrosine. Detectable phenolic structures comprise monophenols such as thymol (an aromatic monoterpene), benzyl alcohols, phenylethanoids (e.g., tyrosol), and the coumarins (e.g., umbelliferone) (Figure 2). The huge class of aromatic acids include benzoic acid and cinnamic acid derivatives with different degree of hydroxylation and methoxylation. Metabolites with vitamin function such as vitamin E (tocopherols) and vitamin K (phylloquinone and menaquinone) represent minor groups of phenolic structures found in food materials. However, tocopherols like α-, β-, γ- and δ-tocopherol are readily detected in biological samples during profiling experiments. More complexly structured monophenolics comprise phenolic diterpenes (e.g., carnosic acid), phenolic amides including the capsaicinoids with capsaicin as well-known representant, the phenolic lipids, e.g., alkylresorcinols, which are commonly found in cereals (wheat, rye, barley and sorghum), but also in certain tree species and bacteria, and finally benzothiazoles which are mentioned in Subsection 3.6.

### 3.2. Polyphenols

**Figure 3:**
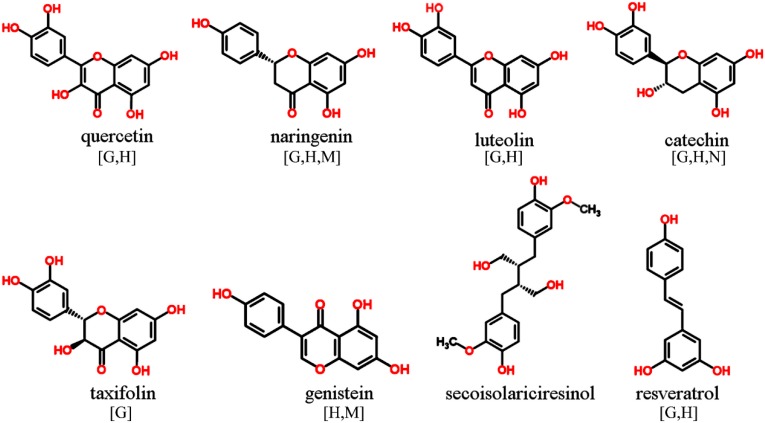
Polyphenols detectable in GC-MS profiling experiments. MS spectra included in publically available databases.

The highly diverse group of plant flavonoids comprise flavonols (e.g., kaempferol and quercetin), flavanones (e.g., naringenin and hesperidin), flavones (e.g., luteolin and apigenin), flavan-3-ols (e.g., catechin and gallocatechin), and flavanonols (e.g., taxifolin) (Figure 3). All these structures, either as aglycon or glycoside, are characterized by a certain number of hydroxy groups which can be silylated. However, due to relatively higher molecular weight of glycosylated polyphenols, the detection and structure elucidation of intact glycosides is preferably achieved on LC platforms, which is also true for hydolyzable tannins. Another closely related group of polyphenols, the phytoestrogens, comprise well-known structures such as isoflavonoids (e.g., genistein and daidzein) commonly found in species of the Fabaceae family, and the lignans (e.g., secoisolariciresinol and pinoresinol) derived from different plant food sources. Also the minor class of stilbenoids, hydroxylated stilbene derivatives such as resveratrol and piceatannol, are readily silylated and detectable if present in appreciable amounts in the sample. In contrast, the important group of anthocyanidins and their glycosylated counterpart, the anthocyanins, show high abundances in fruits and berries but also other plant tissues. These metabolites are normally detected on LC-MS systems not least due to the molecules’ positive charge.

### 3.3. Terpenoids and Sterols

**Figure 4:**
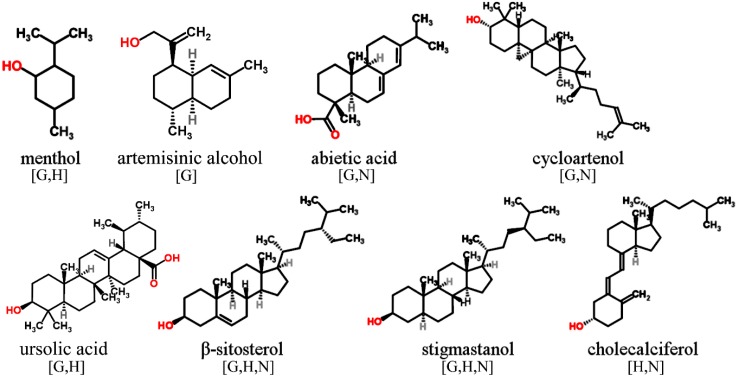
Terpenoids and sterols detectable in GC-MS profiling experiments. MS spectra included in publically available databases.

The terpenoids including the biosynthetically-derived sterols establish a huge class of secondary metabolites which can be found in diverse organisms (Figure 4). Mono- and sesquiterpenes are volatile and lipophilic metabolites commonly found in high abundances in herbs and spices, conifers and other tree species.

The lipophilic phase might preferably be analysed separately prior to GC-MS profiling. However, this step is not always practically feasible, and might result in detection of silylated derivatives of alcoholic mono- and sesquiterpenes, hydroxylated and/or carboxylated di- and triterpenes, and phytosterols including sterols and stanols. Structurally close related is the group of secosteroids including cholecalciferol (vitamin D) and derivatives which are also determined in profiling studies.

### 3.4. N-Containing Cyclic Structures

**Figure 5:**
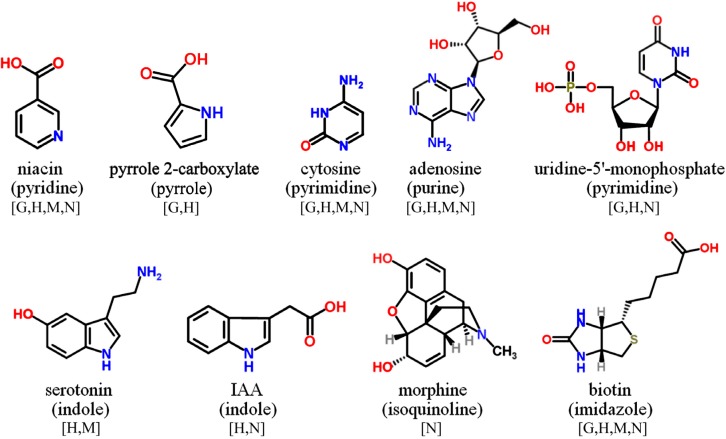
Selected *N*-containing cyclic compounds detectable in GC-MS profiling experiments. Names of the basic heterocyclic structures are given in brackets. MS spectra included in publically available databases.

Alkaloids establish a chemically quite diverse group of basic, nitrogenous secondary metabolites, most of which are characterized by heterocyclic structures (Figure 5). Well-known metabolites include stimulant and/or medicinally significant compounds such as nicotine (based on pyrrolidine/pyridine ring structures), caffeine (a purine), morphine (an isoquinoline), serotonin (an indole), and cocaine (a tropane). Apart from alkaloids, tryptophan-derived indoles establish also the basic ring structures of the plant-hormone related auxines (e.g., indole-3-acetic acid) and several amino acids. Also the cytokinins are *N*-heterocyclic structures with zeatin (a purine) as an important representative compound. Furthermore, most B-vitamins are built up of N-heterocycles comprising detectable structures such as B_3_ niacin and B_6_ pyridoxine (both pyridines), B_7_ biotin (an imidazole), and B_9_ folic acid (a pteridine). Importantly, nitrogenous bases, their nucleosides and phosphorylated nucleotides, which are essential components of RNA and DNA in all organisms, are readily detected in profiling experiments. Cyclic structures comprise pyrimidines (thymine, uracil and cytosine) and purines (adenine and guanine). The vast diversity of N-containing cyclic metabolites from plants does not allow to present all structural classes here. However, it is noteworthy that only a minor fraction of potentially detectable compounds is included in MS libraries of silylated compounds.

### 3.5. O-Containing Cyclic Structures

**Figure 6:**
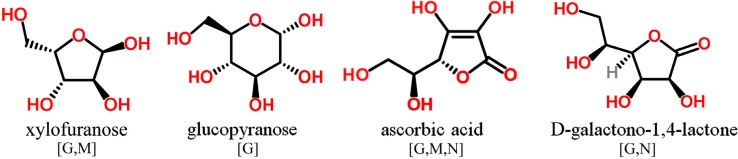
*O*-containing cyclic structures detectable in GC-MS profiling experiments. MS spectra included in publically available databases.

Silylated carbohydrates comprise the largest group of GC-MS detectable oxygen-containing cyclic metabolites. Pentoses and hexoses and oligosaccharides thereof occur in all biological samples showing cyclic structures either as furanoside or pyranoside (Figure 6). Furan structures, in particular lactones derived from sugar acids (e.g., ascorbic acid) but also amino acids are readily determined. This includes the potentially detection of glycosylated compounds such as terpenes, aromatic structures and purines during profiling experiments. Benzopyran structures have already been mentioned in the context of tocopherols (see subsection 3.1).

### 3.6. S-Containing Cyclic Structures

**Figure 7:**
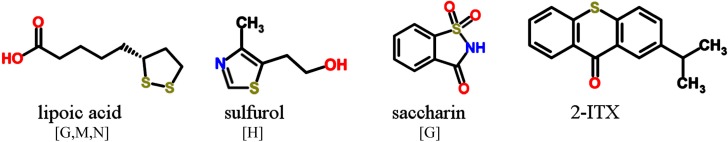
*S*-containing cyclic structures detectable in GC-MS profiling experiments. MS spectra included in publically available databases.

Though nature produces a vast diversity of *S*-containing metabolites, only few cyclic structures have been included in MS libraries of silylated compounds. Those mentioned here both include natural products but also compounds which are not biosynthesized by organisms (Figure 7). Lipoic acid and its derivatives represent dithiolane structures with characteristic disulfide bonds in a pentacyclic structure. Though normally covalently bound in mitochondrial enzyme complexes, these compounds might potentially be detected in profiling experiments. This is also true for thiazoles, *i.e.*, pentacyclic *N*- and *S*-containing structures, heterocyclic compounds such as natural and synthetic benzothiazoles (e.g., the artificial sweetener saccharin), and S-containing polycyclic thioxanthenes used as photoinitiators (e.g., 2-ITX) in paper and packaging materials.

## 4. Case Studies—GC-MS Profiling of Plant Samples

GC-MS is one of the most efficient technology platforms to approach complex mixtures of organic compounds based on a combination of MS database search and the use of calculated RI values. Suitable MS and RI resources have been developed and are well established for the analysis of e.g., essential oil constituents [67,68,69], and investigation of environmental samples using either the comprehensive NIST or Wiley MS libraries or vendor-specific and customized databases. In the case of silylation-based derivatization techniques which cover a broad range of molecular masses and different polarities, the information base regarding phenolic and other cyclic structures, belonging to the complex group of plant secondary metabolites, is rather limited. This is particularly true for higher molecular weight compounds (≥300) as pointed out by Isidorov and Szczepaniak [39]. However, the specificity of molecular structures and thus MS fragmentation patterns in most cases allow for the assignment of distinct compound groups and sub-classes.

In the following Subsection 4.1, Subsection 4.2, Subsection 4.3 and Subsection 4.4, these aspects will be addressed by using examples from the analysis of various plant raw materials and processed plant food to emphasize the applicability of GC-MS profiling for the separation, detection and identification of phenolics and cyclic structures with respect to metabolic phenotyping and quality assessment purposes. Extraction, derivatization and GC-QMS conditions followed procedures for plant samples as described earlier [35,70,71,72].

### 4.1. Fresh Plant Samples: Flavonoids and Derivatives

Flavonoids represent a highly diverse class of polycyclic secondary structures commonly found in the plant kingdom. In addition to their function as pigments for insect attraction, seed dispersal and UV light absorption, flavonoids serve as antioxidants and radical scavengers, in plant signaling and as defense compounds. Chemically, flavonoids are characterized by a C_6_-C_3_-C_6_ flavone skeleton (A-C-B rings) enclosed with oxygen in the 3-carbon bridge (C-ring) between the phenyl groups. The different sub-classes of flavonoid structures include flavones, flavonols, flavanones, flavanols, flavanonols, anthocyanidins, isoflavones, chalcones and neoflavonoids, as reviewed by Tsao and McCallum [73].

Depending on desaturation and oxidation status of the C-ring and moreover, hydroxylation, methoxylation, and/or prenylation patterns of the flavone backbone (for examples refer to Figure 3), EI-based fragmentation is expected to generate quite stable MS fragments with distinct molecular masses, thus providing sufficient identification capability for structure elucidation.

**Figure 8:**
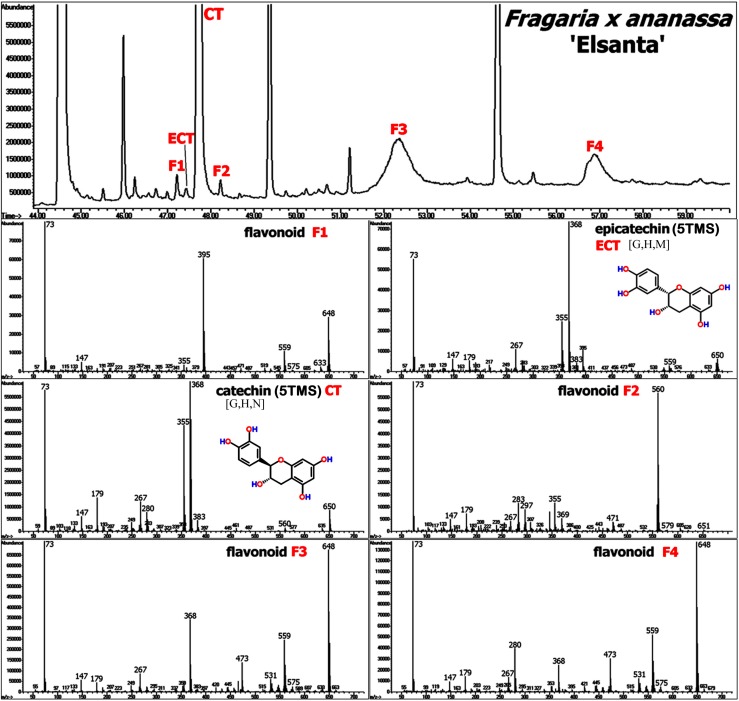
The upper figure shows a chromatogram cut from the analysis of a strawberry crown sample (vegetative tissue) of *Fragaria x ananassa* Duch. cv. “Elsanta”. The MS spectra of commonly found TMS derivatives of polyphenolic structures such as catechin (5TMS) (CT), epicatechin (5TMS) (ECT) and unidentified flavonoid structures (F1 to F4) are depicted in the figures below. MS spectra of CT and ECT can be found in the Golm Metabolome Database [G], Human Metabolome Database [H], MassBank [M], and/or NIST Chemistry WebBook [N].

**Figure 9:**
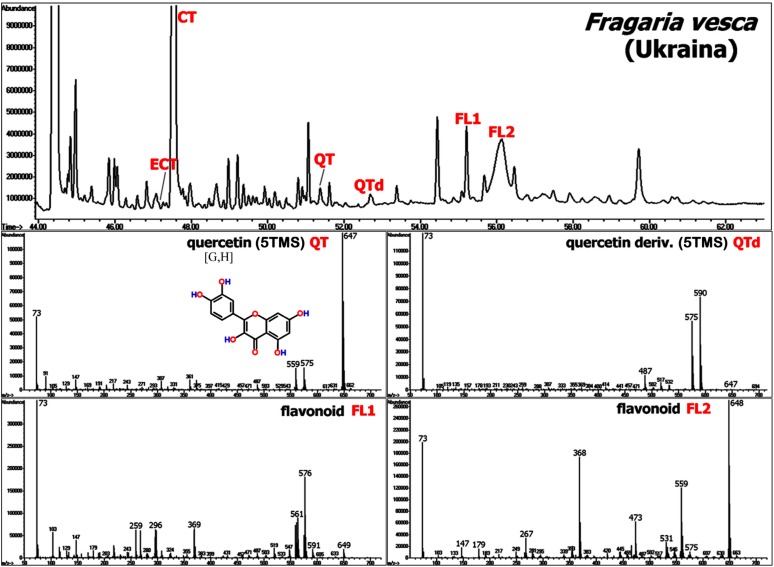
The upper figure shows a chromatogram (TIC), cut from the analysis of a strawberry leaf sample (*Fragaria vesca* L., genotype “Ukraina”). Several MS spectra of TMS derivatives of flavonoids such as quercetin (5TMS) (QT), quercetin derivative (5TMS) (QTd), and other flavonoid structures (FL1 and FL2) are depicted below. CT = catechin (5TMS) and ECT = epicatechin (5TMS). MS spectra of CT, ECT and QT can be found in the Golm Metabolome Database [G], Human Metabolome Database [H], MassBank [M], and/or NIST Chemistry WebBook [N] (see also Figure 8).

Strawberry (*Fragaria × ananassa* Duch.) is renown as a marketable fruit due to its pleasant taste and flavour [74], and its high content of health-beneficial polyphenolic compounds [75,76,77]. Also other plant parts of different *Fragaria* species were shown to contain high levels of phenolic structures as studied in flowers [78], leaves [79,80] and roots [81,82]. Moreover, GC-MS profiling has been recently applied for the characterization of shifts in metabolite pools in leaf and crown tissue of strawberry plants exposed to cold temperatures [70,72,83] in order to identify those compounds uniquely linked to cold acclimation. Published chemical information was related to primary metabolites in the first place, however a large number of secondary structures could be deduced easily based on the available high-resolution MS information from crown [83], and leaf and root samples [72]. Besides cinnamic acid- and benzoic acid-derived structures, vegetative tissue contains reasonable amounts of flavan-3-ols (catechin derivatives) and flavonols as presented in Figure 8 and Figure 9.

### 4.2. Plant-Based Aquafeeds: Phenolic Acids

Fish feeds are formulated from marine (fish meal and oil), animal (e.g., blood meal and poultry by- products), and plant (e.g., starchy grains, protein meals, and oils) feedstuffs, and additional amino acid and micronutrient supplements. Due to limited resources of fishmeal and fish oil and the over-exploitation of wild fish stocks, plant feedstuffs derived from seeds are considered more sustainable, cost efficient and highly valuable as protein ingredients in aquafeeds. On the other hand, seeds contain well-characterized and supposedly unknown phytochemicals (secondary metabolites), which might impair appetite, nutrient utilization, physiology, fish health and growth [84], particularly in carnivorous fishes.

**Figure 10:**
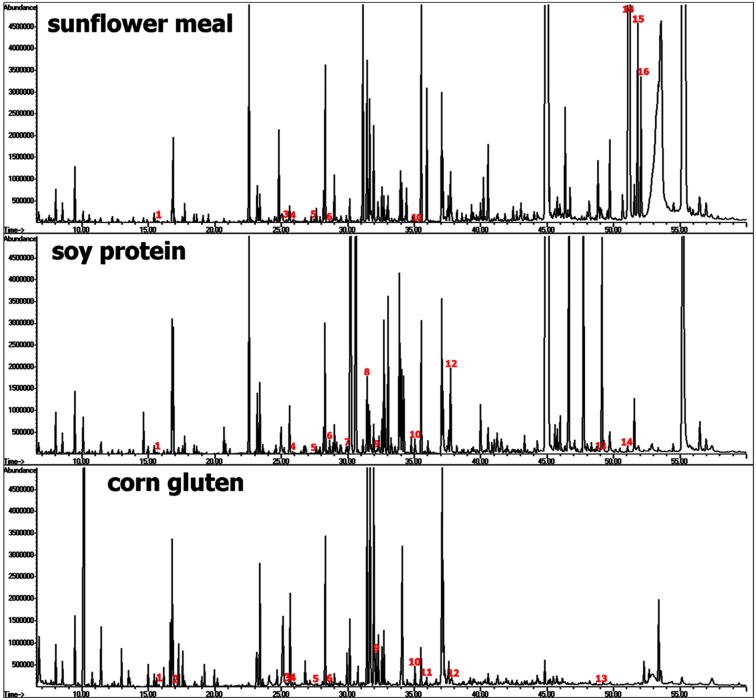
GC-MS-based metabolite profiling for the detection of phenolics and polycyclic structures in plant-derived aquafeed ingredients (sunflower meal, soy protein and corn gluten).

Therefore, these substances are also termed as antinutritional factors (ANF) because of their non-nutrient function [85]. In a recent study, GC-MS-based metabolite profiling was applied in order to gain detailed chemical information about plant derived feedstuffs with regard to nutritious small molecules (carbohydrates, lipids, amino acids and amines) and simultaneously potential ANFs [35], comprising compounds from diverse structure groups such as phenolics, alkaloids, terpenes, and glycosides. Protein-rich seeds from the legume plant family (e.g., soybean and pea) or refined plant ingredients derived from industrial processes after vegetable oil or starch extraction (e.g., sunflower, rapeseed and corn) represent good sources of plant proteins (Figure 10).

**Figure 11:**
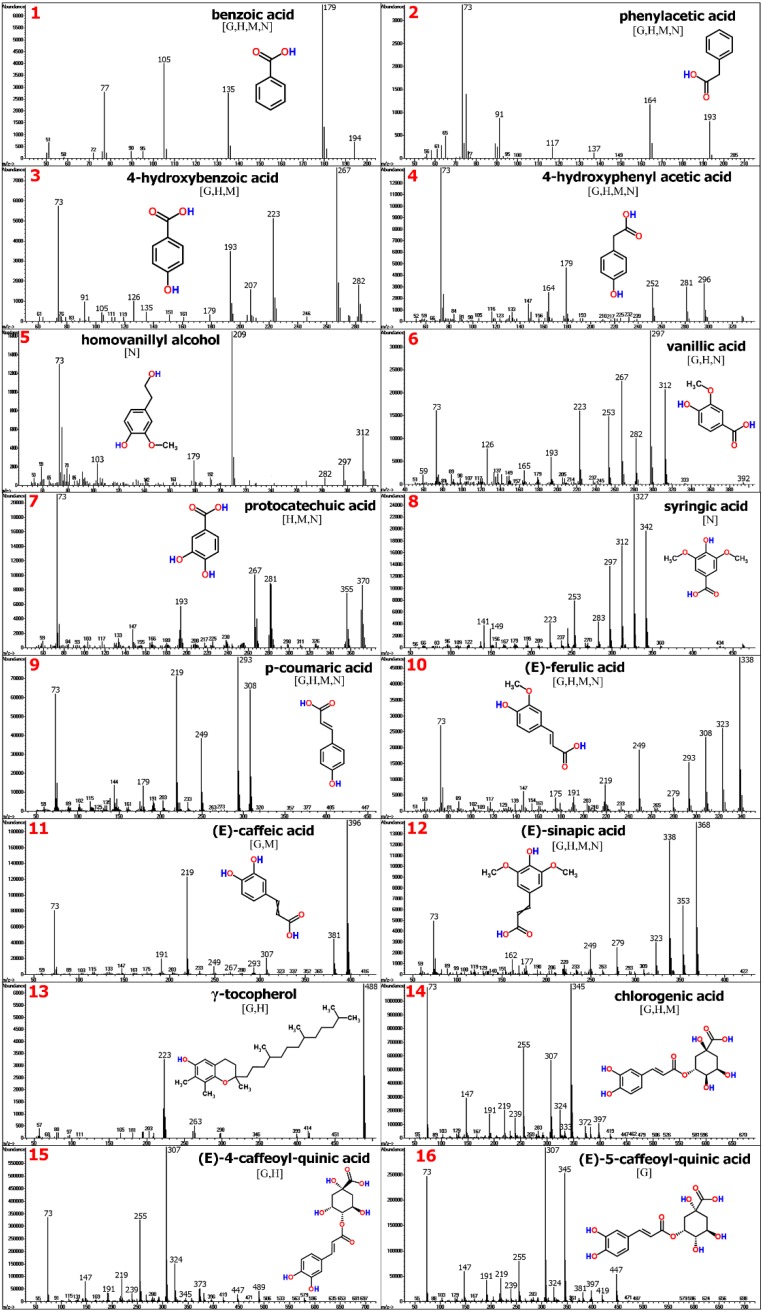
The MS spectra of corresponding monocyclic/polycyclic TMS metabolites (see Figure 10) indicated by nos. **1** to **16**, are depicted in the figures above. MS spectra can be found in the Golm Metabolome Database [G], Human Metabolome Database [H], MassBank [M], and/or NIST Chemistry WebBook [N].

Despite industrial refinement and concentration steps, plant ingredients such as sunflower meal, soy protein concentrate and corn gluten might still contain reasonable amounts of free and bound phenolic ANFs due to interactions with polysaccharides and/or proteins [86], showing a broad spectrum of compounds derived from benzoic acid, cinnamic acid and phenyl ethanol (Figure 11). Relatively high levels of vanillic acid (20–100 mg/kg), syringic acid (40–150 mg/kg), (*E*)-ferulic acid (20–60 mg/kg) and sinapic acid (20–60 mg/kg) were found in soybean meal and thus, underscore the potential ANF content related to non-flavonoid phenolics ranging between 660 to 2000 mg/kg in dehulled beans [87]. In comparison, sunflower seeds are known to contain relatively high levels of mono- and diacylquinic acids [88], which might negatively affect taste and nutrient uptake in humans [89].

Levels of different caffeoylquinic acid structures including chlorogenic acid in sunflower meal (Figure 11) ranged between 600 to 2200 mg/kg and compared well with reported estimates in literature e.g., [90,91].

### 4.3. Cereals: Alkyresorcinols

Cereal grains (wheat, rye, barley and oat) constitute one of the major sources of staple foods for human consumption worldwide due to their nutritious content of carbohydrates, proteins and lipids, also including minerals, vitamins and dietary fibre.

**Figure 12:**
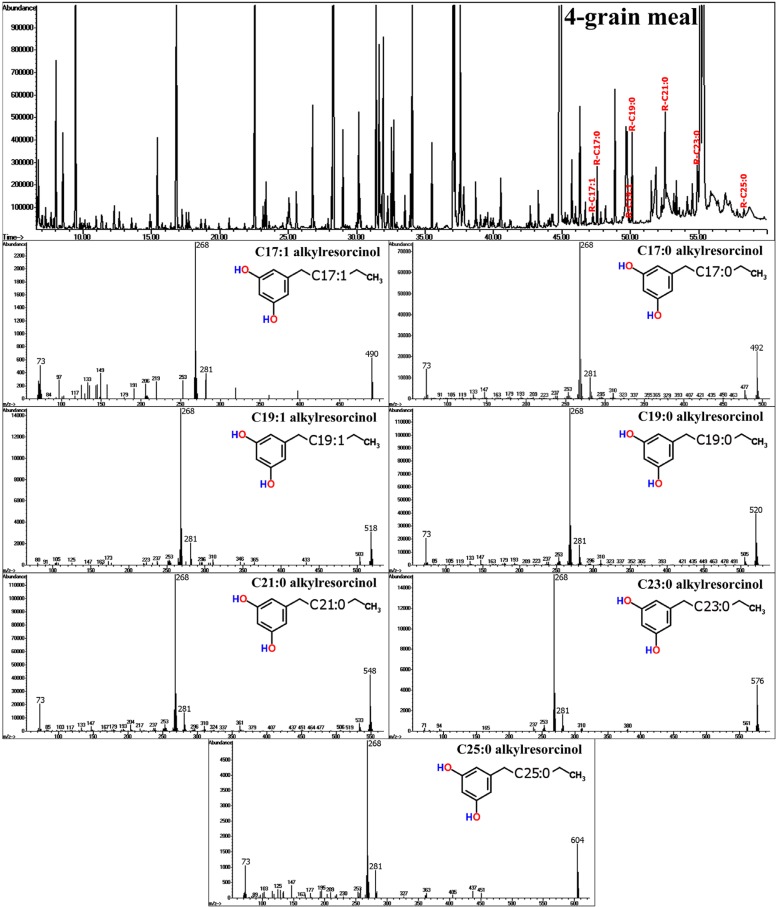
In the GC-MS chromatogram of four-grain meal, commonly occurring alkylresorcinols are indicated. The corresponding MS spectra of C17- to C25- alkylresorcinols are depicted below.

Moreover, recent epidemiological studies have shown that intake of whole grain products is positively linked to prevention of metabolic syndrome, obesity, cardiovascular disease and type 2 diabetes [92]. Despite relatively high concentrations of phenolic antioxidants in fruits and berries, the impact of levels of phenolic compounds in grain products and cereals on human health is underestimated. In other words, based on Western food traditions the intake of health-beneficial plant phenolics is not necessarily and primarily based on the consumption of fruits and vegetables. Cereal products contain relatively high levels of benzoic acid and cinnamic acid derivatives [93], either free or in bound form esterified with cell wall components. Based on data from the multinational HEALTHGRAIN study, wheat, rye and oat grain show comparable levels of phenolics, while concentration levels in barley are somewhat lower [94].

The so-called alkylresorcinols (AR) establish a characteristic sub-class of phenolic compounds found in cereals. ARs are based on a 1,3-dihydroxy-5-alkylbenzene structure being linked with an odd-numbered alkyl or alkenyl chain (C17:0 to C25:0) [95,96,97], and have been suggested to be used as biomarkers for the estimation of whole grain consumption in humans [98]. Due to their unique chemical structure, ARs show distinct EI-MS fragmentation patterns generating a base peak of *m/z* = 268 and a molecular ion (M^+^) peak, depending on the length and degree of saturation of the side chain (Figure 12). These molecular features facilitate the straight-forward detection and identification also in comprehensive GC-MS metabolite profiles. Detected levels of ARs in industrially-processed grain and bakery products were clearly depending on coarseness and declined with the degree of refinement. AR levels in bread ranged from 10 to 300 mg/kg dry weight (DW) and 200 to 300 mg/kg DW in wholemeal bread, while levels in low-processed four-grain meals were estimated at 500 to 650 mg/kg DW, thus corresponding well with results from other studies [46,91,95,96,97].

### 4.4. Olive Oil: Simple Phenolic Structures and Secoiridoids

Olive oil is a vegetable oil produced from fruits of the olive tree (*Olea europaea* L.) and its subspecies. The olive tree is a traditional wood species in Mediterranean countries, the main production region in the world, but olive oil is also produced in Asia, the Americas and Australia. The oil is commonly used in cooking, cosmetics, pharmaceuticals, soaps and as a fuel for oil lamps. Olive oil is considered as a highly-valuable and healthy oil because of its high content of glyceridic-bound monounsaturated fatty acids, mainly oleic acid (C18:1), linoleic acid (18:2) and α-linolenic acid (C18:3). In addition, olive oil contains sterols, triterpenic compounds, aliphatic alcohols and esters, and reasonable amounts of different phenolic structures. Tyrosol and its derivatives, namely oleuropeins and ligstrosides, represent characteristic phenolic structures found in olive exerting health-beneficial effects as reported by the European Food Safety Authority (EFSA) [99]. Other commonly detected phenolic compounds comprise hydroxybenzoic-, hydroxycinnamic- and hydroxyphenylacetic acids, lignans and flavonoids [91]. Phenolic patterns found in olive oil can be used to study regional and varietal differences [100], and effects of oil production and processing [101]. Different approaches towards extraction and chromatographic separation have been described [102,103], also including derivatization methods following GC-MS for the analysis of olive and other vegetable oils [41,104,105,106].

The quality of olive oils is mainly based on extraction and processing conditions, and distinct quality parameters such as acidity, taste and flavour characteristics. According to the classification system of the International Olive Council, oils can be divided into extra-virgin, virgin and the chemically-treated refined oils which all are originally obtained by pressing, and the solvent-extracted pomace oils. Depending on the physical and chemical extraction and processing steps utilized, the content and composition of phenolics shows a high degree of variation. Extra virgin oils often show two to three times higher levels of phenolic compounds compared to refined oil qualities [91].

**Figure 13:**
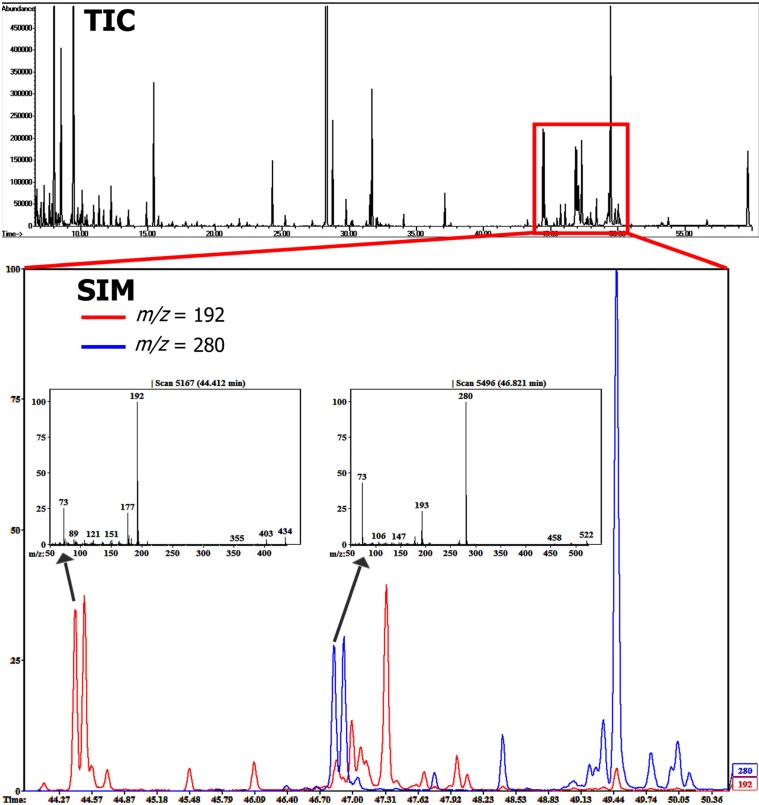
GC-MS chromatogram of the polar (phenolic) fraction of an example extra- virgin olive oil. The elution region of secoiridoids is highlighted, zooming in to a SIM chromatogram plot indicating separation patterns of ligstroside (*m/z* = 192) and oleuropein (*m/z* = 280) derivatives. MS spectra included in publicly available databases: [G]—Golm Metabolome Database; [H]—Human Metabolome Database; [M]—MassBank; [N]—NIST Chemistry WebBook.

**Table 2:** Lowest, highest and mean levels of phenolic and cyclic structures detected in commercial olive oils (mg/kg FW). Retention time (RT) and retention index (RI) are based on an apolar HP-5MS column. The relative intensity (in %) of mass ions is shown in parenthesis. MS spectra included in publicly available databases (DB): [G] Golm Metabolome Database; [H] Human Metabolome Database; [M] MassBank; [N] NIST Chemistry WebBook.

RT	RI	Compound	Masses	DB	Mean	Low	High
15.05	1238	phenylethyl alcohol	M^+^ 194(1), 73(100), 103(81), 179(68), 105(24)	[N]	0.19	0.04	0.82
15.40	1249	3,5-dimethylphenol	M^+^ 194(39), 179(100), 194(67), 105(16)	[N]	0.28	0.03	1.45
15.64	1257	benzoic acid	M^+^ 194(4), 179(100), 105(79), 135(62), 77(56)	[G,H,M,N]	0.44	0.02	1.32
17.13	1306	phenylacetic acid	M^+^ 208, 73(100), 164(21), 193(9), 137(3)	[G,H,M,N]	0.26	0.04	0.87
17.80	1329	catechol	M^+^ 254(100), 239(30), 151(20), 136(15), 166(13)	[G,H,M,N]	5.15	0.45	28.52
20.34	1419	hydrocinnamic acid	M^+^ 222(24), 104(100), 207(46), 91(26), 132(4)	[G,H,M,N]	0.60	0.01	3.25
21.62	1466	hydroxybenzoic acid	M^+^ 282(48), 73(100), 267(18), 179(14), 193(10)	[G,H,M]	0.47	0.03	2.36
21.82	1473	(*E*)-isoeugenol	M^+^ 236(42), 206(100), 73(21), 221(19), 179(11), 103(6)	[N]	0.81	0.02	4.65
22.86	1513	salicylic acid	M^+^ 282, 73(100), 267(92), 135(10), 193(5)	[G,H,N]	0.17	0.01	0.49
23.83	1551	syringaldehyde	M^+^ 254(45), 224(100), 73(79), 209(45), 239(33)	[N]	0.04	0.03	0.07
24.26	1569	*p*-tyrosol	M^+^ 282(19), 179(100), 267(13), 193(12)	[H]	7.02	1.47	18.32
25.23	1608	ligstroside deriv.	M^+^ 192(100), 177(67), 179(24), 193(19)	–	4.76	0.04	18.91
25.62	1624	methyl homovanillic acid	M^+^ 268(55), 73(100), 238(75), 209(46), 253(30)	–	0.05	0.02	0.14
25.95	1639	vanillin	M^+^ 253(71), 223(100), 73(29), 238(22), 165(12)	[N]	1.28	0.03	6.12
27.45	1703	homovanillyl alcohol	M^+^ 312(33), 73(100), 209(94), 103(22), 179(16)	[N]	0.61	0.01	3.05
28.49	1749	phloretic acid	M^+^ 308(52), 73(100), 219(71), 293(65), 249(53)	[N]	0.11	0.04	0.46
28.63	1755	vanillic acid	M^+^ 312(24), 267(49), 297(44), 282(34), 253(28)	[G,H,N]	0.43	0.02	2.26
28.76	1761	hydroxytyrosol	M^+^ 370(44), 267(100), 193(19), 179(11)	–	1.47	0.27	5.35
29.77	1807	oleuropein deriv.	M^+^ 340(13), 73(100), 280(96), 193(40), 179(14)	–	7.19	0.17	35.98
30.75	1853	*p*-coumaric acid	M^+^ 308(35), 73(100), 293(53), 219(42), 249(23)	[G,H,M,N]	0.04	0.02	0.06
31.44	1887	syringic acid	M^+^ 342(24), 327(100), 73(67), 312(64), 297(58)	[N]	0.04	0.01	0.07
32.11	1919	(*Z*)-ferulic acid	M^+^ 338(49), 73(100), 308(43), 323(37), 249(33)	[G,N]	0.08	0.02	0.26
32.14	1921	(*E*)-coniferaldehyde	M^+^ 279(27), 73(100), 248(56), 218(49), 232(14)	[G]	0.06	0.04	0.15
43.86	2596	ligstroside deriv.*	192(100), 177(26), 179(8)	–	0.13	0.05	0.60
44.12	2613	ligstroside deriv.*	192(100), 177(22)	–	0.17	0.04	0.49
44.41	2633	ligstroside aglycone (aldehydic form I)	M^+^ 434(5), 192(100), 177(19), 179(7), 403(3)	–	4.42	0.07	25.91
44.57	2644	ligstroside aglycone (aldehydic form II)	M^+^ 434(5), 192(100), 177(23), 179(7), 403(3)	–	0.59	0.07	3.15
45.48	2706	ligstroside aglycone deriv.*	192(100), 177(15), 179(5)	–	0.34	0.03	1.51
46.08	2749	ligstroside aglycone deriv.*	192(100), 177(22), 179(5)	–	0.49	0.04	1.96
46.39	2770	oleuropein deriv.*	280(100), 193(35), 179(14)	–	0.09	0.02	0.36
46.66	2790	oleuropein deriv.*	280(100), 192(74), 177(16)	–	0.08	0.02	0.27
46.92	2809	oleuropein aglycone (aldehydic form)	M^+^ 522(3), 280(100), 193(18), 179(4), 267(3)	–	3.48	0.07	20.45
47.00	2814	ligstroside deriv.	M^+^ 492(3), 192(100), 177(29), 280(13), 209(4), 461(2)	–	0.81	0.03	4.66
47.08	2820	ligstroside deriv.	M^+^ 492(2), 192(100), 177(30), 461(21), 209(14), 280(9)	–	0.52	0.03	2.91
47.32	2837	ligstroside deriv.	M^+^ 492(1), 192(100), 177(16), 179(5), 209(2), 280(1)	–	2.57	0.07	14.28
47.67	2863	ligstroside deriv.*	192(100), 177(18), 355(10), 179(8)	–	0.23	0.03	1.15
47.77	2870	oleuropein deriv.*	280(100), 193(26), 179(4)	–	0.25	0.01	1.22
47.91	2881	ligstroside deriv.*	192(100), 177(38), 179(19)	–	0.08	0.02	0.26
47.98	2886	ligstroside deriv.*	192(100), 177(16), 179(6)	–	0.46	0.03	2.27
48.07	2893	ligstroside deriv.*	192(100), 177(18), 179(9)	–	0.20	0.02	1.01
48.40	2918	oleuropein deriv.*	280(100), 193(23), 179(6), 267(4), 519(4)	–	0.73	0.04	3.82
48.63	2935	oleuropein deriv.*	280(100), 193(44), 192(41)	–	0.05	0.01	0.08
49.07	2968	oleuropein deriv.*	280(100), 193(28), 179(8), 355(7)	–	0.14	0.02	0.66
49.13	2973	oleuropein deriv.*	280(100), 193(28), 179(14)	–	0.10	0.02	0.37
49.22	2979	oleuropein deriv.	M^+^ 580(2), 280(100), 193(21), 179(5), 267(4)	–	0.33	0.03	1.80
49.27	2984	oleuropein deriv.	M^+^ 549(5), 280(100), 193(27), 179(8), 267(4)	–	0.31	0.03	1.68
49.35	2989	oleuropein deriv.	M^+^ 551(2), 280(100), 193(21), 179(4), 519(1)	–	0.88	0.04	4.94
49.46	2998	oleuropein deriv.	M^+^ 551(2), 280(100), 193(19), 179(4), 519(1)	–	6.32	0.08	36.78
49.79	3023	oleuropein deriv.	M^+^ 551(2), 280(100), 193(18), 179(5), 267(4)	–	0.47	0.02	2.62
49.98	3038	oleuropein deriv.*	280(100), 355(30), 193(20), 368(4)	–	0.30	0.02	1.60
50.04	3043	oleuropein deriv.*	280(100), 193(18), 179(5), 355(2)	–	0.64	0.04	3.43
50.15	3051	oleuropein deriv.*	280(100), 193(17), 179(7), 267(5)	–	0.23	0.01	1.16
51.78	3182	luteolin	M^+^ 559(100), 73(54), 487(8), 272(7)	[G,H]	0.05	0.01	0.09
53.26	3306	pinoresinol	M^+^ 502(59), 223(100), 73(75), 209(56), 235(43)	–	0.08	0.02	0.16
53.34	3313	β-sitosterol	M^+^ 486(25), 129(100), 357(97), 396(92), 73(63), 381(40)	[G,H,N]	0.12	0.03	0.24
53.76	3348	acetoxipinoresinol	M^+^ 560(18), 276(100), 245(53), 73(37), 209(34), 261(20)	–	1.06	0.04	5.40
55.59	3509	uvaol	M^+^ 496(89), 216(100), 73(58), 203(51), 188(25), 161(24)	–	0.32	0.02	0.96
56.65	3607	oleanolic acid	M^+^ 585(6), 203(100), 73(53), 320(35), 189(33), 482(24)	[G,H]	1.36	0.05	3.77
		**Total Phenolics**			**59.96**	**4.22**	**286.99**
		oleuropein structures			21.61	0.72	117.21
		ligstroside structures			15.78	0.52	79.08
		tyrosols			8.49	1.74	23.67
		phenolic acids			2.69	0.36	11.56
		alcohols			7.04	0.58	38.49
		aldehydes			1.37	0.10	6.34
		lignans			1.13	0.07	5.56
		flavonoids			0.05	0.03	0.09

* no molecular peak detected; – no database MS spectrum available

As an effect of oil processing, refined oils generally show higher abundance of simple tyrosol structures due to the degradation of oleuropeins and ligstrosides. On the other hand, oleuropein and ligstroside content is generally much higher in extra-virgin and virgin oils, not uncommonly exceeding levels above 300 mg/kg. Based on a quality screening of different commercially available olive oils and olive extracts, variations in phenolic metabolites could easily be characterized using liquid-liquid extraction techniques and compound derivatization. Due to EI fragmentation patterns, secoiridoid aglyca could be easily traced using selected ion monitoring (SIM) plots depicting the distinct MS base peaks related to ligstroside (*m/z* = 192) and oleuropein (*m/z* = 280) structures (Figure 13). A total of 55 phenolic metabolites and three other cyclic structures could be detected, of which 28 compounds were tentatively identified based on a combination of MS database search and retention index values (Table 2).

## 5. Conclusions

GC-MS is frequently applied to characterize the chemical complexity of analytical samples based on its separation and identification capacity. Recent developments in GC-MS technology have facilitated global metabolomics approaches in order to approach biological functions and perturbations of biological systems, and for diagnostics and quality assessment purposes. However, one should be aware of the limitations of global GC-MS metabolite profiling. Processing, automated sample handling, and analysis conditions need to be strictly defined and controlled in order to minimize data variation and allow for quantitative calculations. When using standard protocols which are adapted to cover a broad range of biochemical structures, single metabolites or groups of compounds might be discriminated due to generalized compound extraction and derivatization conditions and thus, negatively affect compound recovery rates. Moreover, GC-MS analysis of highly complex mixtures of derivatized metabolites might impair separation and detection capacity with regard to the level of confidence in compound identification due to co-eluting peaks and similarity of MS spectra. For determination of absolute metabolite concentrations, the use of standard compounds is required, otherwise targeted methods need to be applied for the proper quantitation of compounds of interest. Despite limitations in GC-MS with respect to the mass range and polarity of metabolites, the utilization of derivatization techniques and automation technology have extended the range of separable and detectable compounds in high-throughput profiling experiments. Beside the qualitative and quantitative analysis of trimethylsilyl derivatives of highly abundant compounds found in plant samples such as sugars, amino acids and polyols, instrument sensitivity and resolution also allows for the successful detection of minor constituents such as plant secondary metabolites. Even though mass spectral information about monophenolic, polyphenolic and other cyclic compounds in MS libraries is limited, structure-specific MS fragmentation patterns enable to trace and identify low-concentration metabolites, often based on and in combination with published MS data from targeted GC-MS analyses. Current limitations in MS-based metabolomics due to the relatively small number of compounds included in MS databases, in particular secondary metabolites, and hurdles in compound identification, might be overcome by on-going and future efforts. These include *in silico* derivatization, retention indices and mass spectra matching [107], *in silico* enzymatic synthesis of biochemical compounds for non-targeted metabolomics [108], and endeavours such as web-based and shared collections of experimental metabolomics datasets, MS spectra and RI values for the processing and interpretation of GC-MS data [62]. Based on experimental data from own research, the present review has emphasized the capabilities of GC-MS to deduce chemical information on phenolics and cyclic compounds found in complex mixtures of plant metabolites.
